# A series of unfortunate events: the forgotten botanist and the misattribution of a type collection

**DOI:** 10.3897/phytokeys.109.28144

**Published:** 2018-09-19

**Authors:** Eric J. Tepe

**Affiliations:** 1 Department of Biological Sciences, University of Cincinnati, Cincinnati, Ohio 45221, USA University of Cincinnati Cincinnati United States of America

**Keywords:** Ecuador, Juan Tafalla, Peru, Real Expedición, Hipólito Ruiz and José Pavón, Scientific expeditions

## Abstract

In 1777, Spain sent The Royal Expedition to Peru and Chile, commonly referred to as the “Ruiz and Pavón Expedition”, to document the flora of this richly diverse part of its colonies. The Expedition was active in the New World for 38 years and was led by Hipólito Ruiz and José Pavón, who collected in Peru and Chile for the first 11 years before returning to Spain to work on publication of the *Flora Peruviana, et Chilensis*. Work on the ground for the reminder of the project in Peru and later Ecuador, was continued by Juan Tafalla and Juan Manzanilla. With few exceptions, all collections from the Expedition including types, have conventionally been attributed to Ruiz and Pavón despite the fact that Tafalla and Manzanilla were responsible for a large part of the collections. The purpose of this note is to bring to light the important but often overlooked figure of Juan Tafalla, to highlight his accomplishments as a collector and to increase precision in botanical nomenclature by giving him due credit by correctly attributing collections.

## Abstract (es)

En 1777, España mandó la Real Expedición Botánica al Virreinato del Perú, más conocida como la “expedición de Ruiz y Pavón,” con el fin de documentar la flora de esta parte de sus colonias notable por su diversidad. La expedición continuó en el nuevo mundo por 38 años y fue dirigida por Hipólito Ruiz y José Pavón durante los primeros 11 años, quienes recolectaron en Perú y Chile antes de volver a España para trabajar en la publicación de la *Flora Peruviana, et Chilensis*. El proyecto fue continuado en Perú, y luego Ecuador, por Juan Tafalla y Juan Manzanilla. Con pocas excepciones, todas las colecciones de la expedición, incluyendo tipos, han sido atribuidos a Ruiz y Pavón a pesar de que Tafalla y Manzanilla son responsables de una gran cantidad de las colecciones. El propósito de esta nota es traer a la luz la figura importante pero frecuentemente ignorada de Juan Tafalla, destacar sus logros como colector, y mejorar la precisión en la nomenclatura botánica.

## Introduction

**Figure 1.**
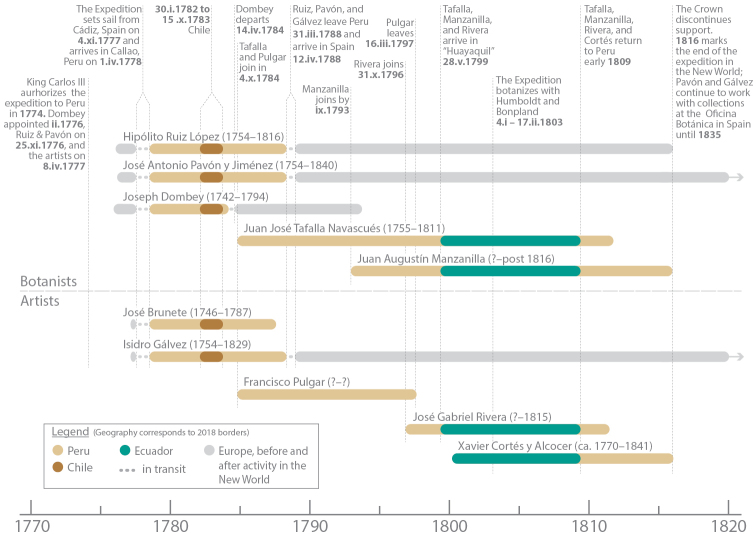
Timeline of participants during the New World portion of the *Real Expedición Botánica al Virreinato del Perú* (1778–1816). Dates are day, month in Roman numerals and year. The years following participants' names are birth–death. Bars in the timeline are limited to individuals' active participation in the project. Dates from Steele (1964); Estrella (1989); Adanero Oslé (2011); Pupulin (2012a); A. Wulf (pers. comm.).

The following is a correction of a previously published revision of Solanumsect.Herpystichum (Tepe and Bohs 2011) and a historical note intended to help future authors avoid making the same mistake. In that work, I repeated Michel Felix Dunal’s attribution of the type collection of *Solanumtrifolium* Dunal, a species endemic to the central Andes of Ecuador, to Ruiz and Pavón (Tepe and Bohs 2011). Ruiz and Pavón, however, never collected in Ecuador and, in fact, by the time that collection was made in August 1804, the pair had already been back in Spain for over six years (Figure 1). The type specimen of this species was actually collected by a little-known botanist and collaborator of Ruiz and Pavón, Juan José Tafalla y Navascués (sometimes spelled Nabasques) and his assistant, Juan Manzanilla (Estrella 1989; Goicoechea Marcaida 1989; Adanero Oslé 2011).

This error stems from several interrelated causes. Spain organized an effort in the late 18^th^ century to document the flora of Peru and, although a number of people would conduct the project on the ground in the New World, the entire enterprise is frequently referred to as “The Ruiz and Pavón Expedition.” A second reason lies with the ownership and control that Ruiz and Pavón exerted over the entire enterprise, even after their return to Spain. And thirdly, confusion over the collectors of specimens was further confounded by the manner in which specimens collected during the Expedition were initially labeled, later intermixed and, eventually, disseminated. All of this has contributed to largely pushing Juan Tafalla, one of the most prolific early collectors of Neotropical flora, into obscurity.

The story of Tafalla, the botanist, begins with the establishment of the *Real Expedición Botánica al Virreinato del Perú* (1777–1816), more commonly known, as mentioned above, as “The Ruiz and Pavón Expedition” (Steele 1964). In 1777, the Spanish Crown sent Hipólito Ruiz López, José Antonio Pavón Jiménez, French botanist Joseph Dombey, along with artists José Brunete and Isidro Gálvez, to the Viceroyalty of Peru, on what was initially planned to be a four-year effort to produce a comprehensive and beautifully-illustrated flora of the territory. The Viceroyalty, during the time of the Expedition (“*Peruvia*” on labels and in subsequent works), was much larger than the current Peruvian territory and encompassed modern day Peru, along with parts of Bolivia, Brazil, Chile and Ecuador. The job turned out to be much larger than expected – not surprising for what we now recognize as one of Earth’s biodiversity hotspots (Myers et al. 2010; Ulloa Ulloa et al. 2017) – and, instead of four years, the venture was active in the New World for 38 years (Steele 1964).

Having already invested more time than anticipated in the New World, the ageing polymath Dombey retired from the Expedition and departed from Peru for Europe in 1784 (Steele 1964). As a replacement for Dombey, and planning for long-term productivity, Spain appointed Juan Tafalla to apprentice under Ruiz and Pavón and another artist, Francisco Pulgar, to work under Brunete and Gálvez. These newcomers were initially not well received by the original members of the Expedition and, throughout much of Ruiz’s journals, they are referred to as the “agregados,” or attachés (Steele 1964). This lack of recognition by name would last for years and Ruiz even requested a replacement for Tafalla (Estrella 1989; Ruiz 2007). Ruiz, however, ultimately came around and acknowledged Tafalla’s careful work and dedication to the Expedition (Estrella 1989; Ruiz 2007). Eventually, Ruiz honored Tafalla with the dedication of the genus *Tafalla* Ruiz & Pav. (now a synonym of *Hedyosmum* Sw., Chloranthaceae) stating that the genus was “…dedicated to our esteemed disciple…” (Ruiz and Pavón 1794; author’s translation).

Tafalla was appointed as head of the New World portion of the Expedition when Ruiz and Pavón received orders on 12 Sept 1787 to return to Spain and begin working towards publishing the Flora (Steele 1964; Ruiz 2007). Although he was now in charge, Tafalla never gained independence from Ruiz and Pavón, who considered him their subordinate throughout the project and to whom they frequently sent requests for additional details to refine their work. Tafalla was productive, despite limited financial support and, ultimately, the Crown provided the Expedition with another botanist, Juan Augustín Manzanilla, in 1793; other new additions to the project came in 1796 with the artist José Gabriel Rivera and, in 1800, the artist Xavier Cortés y Alcocer (Steele 1964; Adanero Oslé 2011; Pupulin 2012a). Tafalla went on to lead the Expedition in Peru for another eleven years until, upon receiving orders from the Crown, moving on to Ecuador (at the time, the *Real Audiencia de Quito*). The team collected in the coastal plains and foothills of the Andes from 1799 to 1803 before switching their focus to the southern Andes of Ecuador and to the investigation of *Cinchona* L. (Rubiaceae), the source of the antimalarial quinine (Steele 1964). While in Ecuador, members of the Expedition met and botanized with Alexander von Humboldt and Aimé Bonpland from 4 January to 17 February 1803 (A. Wulf, pers. comm.), and Humboldt wrote favorably about Tafalla and his team (Estrella 1989; Adanero Oslé 2011).

Meanwhile, shortly after their return to Spain, Ruiz and Pavón had established the *Oficina Botánica* or *Oficina de la Flora Americana* in Madrid, where they studied specimens and prepared their works for publication (Steele 1964; Rodríguez Nozal 1995). During this time, Tafalla continued to collect and to send a large number of specimens, seeds, illustrations, descriptions of new species and other items of interest to Ruiz and Pavón. Between 1788 and 1815, Tafalla sent no fewer than 101 shipments which, upon arrival, were mixed with previous collections and published in the “Flora Peruviana” and other works (Ruiz and Pavón 1794, 1798a, 1798b, 1799, 1802). Tafalla received acknowledgement for is contribution to these works, but not co-authorship (Adanero Oslé 2011). In 1799, shortly after arriving in Ecuador, Tafalla changed his labeling of plants from “*F. P.*” for *Flora Peruviana* to “*F. H.*” for *Flora Huayaquilensis* (Adanero Oslé 2011). Even though Ruiz and Pavón ignored this new labeling system and continued to group Tafalla’s collections with the rest, this change suggests that Tafalla saw this work as an independent project. During his time in Ecuador, Tafalla produced a nearly complete manuscript, which remained unpublished and was only rediscovered by the medical doctor and science historian Eduardo Estrella in 1985, who published both the descriptions and the illustrations (Tafalla Navascués 1989, 1991). Several authors suggest that the independent publication of Tafalla’s *Flora Huayaquilensis* during his lifetime may have been suppressed (Herrera 1937; Estrella 1989). The fact that his work remained unpublished for centuries is not only a historic oversight, but also had the tragic consequence that numerous Ecuadorian species, although Tafalla was likely the first European to see most of them, were ultimately described by botanists from Germany, France and England etc. (e.g. Bonpland et al. 1815; Don 1830) – some even based on Tafalla’s collections – while Tafalla authored none.

The specimen labels from the Expedition are typical of the era and, when not missing, bear minimal information. A subset of specimens has labels with locality information or collection dates, but, with the exception of some of Dombey’s labels, very few identify the collector. To make matters worse, the identity of the collector is further obscured by the gradual and questionable way in which specimens were disseminated. Following Ruiz’s death in 1811, Pavón began selling specimens to collectors throughout Europe, using the profits to maintain operations of the *Oficina Botánica* (Steele 1964; Miller 1970; Rodríguez Nozal 1994, 1995). For example, Pavón sold at least 15,894 specimens to the renowned British collector and prominent member of the Linnean Society of London, Aylmer Bourke Lambert (many of these specimens are now in the collections of B, BM, FL, G, K, L, P, US and other herbaria.; Rodríguez Nozal 1994). These specimens, having come from Pavón, were frequently attributed to him, regardless of where and by whom they had been collected. Many species were described from these purchased specimens and most of the type collections were attributed to Ruiz and Pavón or merely to Pavón. The rare exceptions were Ecuadorian species described by David Don who attributed two types to Tafalla (Don 1830). In addition to these, Tafalla has also been recognized as the type collector of several additional species in more recent revisions and floristic studies (e.g. Harling 1995; Delprete 1999; Pupulin 2012a, b; Morales and Zamora 2017). On the other hand, authors who are aware of Tafalla and his collections, often assume that all of his collections are Ecuadorian in origin (e.g. O’Leary 2015), even when that would place a specimen well outside a species’ normal range. Of the 27 years that Tafalla spent collecting in South America, 17 were spent collecting in Peru, so a great many of his collections are certainly from Peru. Unfortunately, because of the minimalistic nature of the labels, it is often impossible to know whether the Peruvian specimens were collected by Ruiz and Pavón or by Tafalla, and it is likely that many of the Expedition’s collections from Peru that are attributed to Ruiz and Pavón were, in fact, collected by Tafalla or Tafalla and Manzanilla.

Juan Tafalla deserves recognition for having explored Ecuador and Peru more extensively and more deeply than any other Spanish botanist of his time (Adanero Oslé, 2011), as well as being one of the most prolific collectors of his time and a major contributor to the Ruiz and Pavón Expedition. A small step toward making amends for this historic oversight is to accurately acknowledge Tafalla for his collections. In that spirit, the following is a correction to the type information of *Solanumtrifolium* published by Tepe and Bohs (2011), along with the addition of a recently discovered isotype at OXF.

### Solanum trifolium

Taxon classificationPlantaeSolanalesSolanaceae

Dunal, DC. Prodr. 13(1): 68. 1852.

#### Type.

Ecuador. “In Peruviæ provinciâ Huyaquil” Aug 1804 (fl), J. Tafalla & J. Manzanilla s.n. (holotype: G [G00080145!]; photos of holotype [F neg. 8591]: F [F–651206!], MO [MO–1691471!], NY!; isotypes: MA [MA–747193!], OXF [OXF–00006573!]; possible isotype: G-DC [G00144594!]).

## Supplementary Material

XML Treatment for Solanum trifolium

## References

[B1] Adanero Oslé C (2011) Juan Tafalla, botánico navarro, 200 años después. Revista de la Societat Catalana d'Història de la Farmàcia 6: 23–32.

[B2] Bonpland A von Humboldt A Kunth KS (1815) Nova Genera et Species Plantarum. Theatrum orbis terrarum, Amsterdam.

[B3] Delprete PG (1999) Rubiaceae-Condamineae. In: Harling GW Andersson L (Eds) Flora of Ecuador, vol.62. University of Göteborg, Göteborg, 5–53.

[B4] Don D (1830) Description of the new genera and species of the Class Compositae belonging to the flora of Peru, Mexico, and Chile. Transactions of the Linnean Society of London 16(2): 169–303. 10.1111/j.1095-8339.1829.tb00136.x

[B5] Estrella E (1989) Introducción histórica: La expedición de Juan Tafalla a la Read Audiencia de Quito (1799–1808) y la “Flora Huayaquilensis.” In: Tafalla Navascués JJ (Ed.) Flora Huayaquilensis: sive, Descriptiones et icones plantarum Huayaquilensium secumdum systema Linnaeanum digestae. Tomus I. falla. ICONA and Real Jardín Botánico, Madrid, XIII–CVI.

[B6] Goicoechea Marcaida A (1989) Juan José Tafalla y Nabasques, botánico olvidado de la Ilustración. Principe de Viana 50: 641–648.

[B7] Harling G (1995) The genus *Jungia* L. fil. (Compositae-Mutisieae). Acta Regiae Societatis Scientiarum et Litterarum Gothoburgensis. Botanica 4: 1–133.

[B8] Herrera F (1937) Juan Tafalla: Ilustre botánico español, primer catedrático titular de fitografía en la Universidad de San Marcos. Revista de Ciencias 422: 47–60.

[B9] Miller HS (1970) The Herbarium of Aylmer Bourke Lambert: Notes on Its Acquisition, Dispersal, and Present Whereabouts. Taxon 19(4): 489–553. 10.2307/1218947

[B10] Morales JF Zamora N (2017) A synopsis of Aspidosperma (Apocynaceae) in Mexico and Central America with a taxonomic clarification of Aspidosperma cruentum and a new cryptic species. Phytoneuron 68: 1–13.

[B11] Myers N Mittermeier RA Mittermeier CG da Fonseca GAB Kent J (2010) Biodiversity hotspots for conservation priorities. Nature 403(6772): 853–858. 10.1038/35002501 10706275

[B12] O’Leary N (2015) Tipificaciones en especies sudamericanas de *Hedeoma*, *Lepechina* y *Stachys* y nuevo sinónimo en *Lepechina*. Boletín de la Sociedad Argentina de Botánica 50: 217–220.

[B13] Pupulin F (2012a) The Orchidaceae of Ruiz & Pavón's “Flora Peruviana et Chilensis”. A taxonomic study. I. Anales del Jardín Botanico de Madrid 69(1): 21–79. 10.3989/ajbm.2295

[B14] Pupulin F (2012b) The Orchidaceae of Ruiz & Pavón’s “Flora Peruviana et Chilensis”. A taxonomic study. II. Anales del Jardín Botánico de Madrid 69: 143–186.

[B15] Rodríguez Nozal R (1994) Las colecciones americanas generadas por las expediciones botánicas de la España ilustrada: Un análisis de su dispersión LLULL 17: 403–436.

[B16] Rodríguez Nozal R (1995) La Oficina Botanica (1788–1835): Una institución dedicada al estudio de la flora americana. Asclepio 47(2): 169–183. 10.3989/asclepio.1995.v47.i2.442 11625887

[B17] Ruiz H (2007) Relación del viaje hecho a los reinos del Perú y Chile por los botánicos y dibujantes enviados por el Rey para aquella expedición, extractada de los diarios por el orden que llevó en éstos su autor. In: Rodríguez Nozal R, González Bueno A (Eds) Consejo Superior de Investigaciones Científicas, Madrid.

[B18] Ruiz H Pavón JA (1794) Flora peruvianae, et chilensis prodromus. Typis Gabrielis de Sancha, Madrid. http://bibdigital.rjb.csic.es/ing/FichaLibro.php?Libro=106

[B19] Ruiz H Pavón JA (1798a) Flora peruviana, et chilensis, Tomus 1. Typis Gabrielis de Sancha, Madrid. http://bibdigital.rjb.csic.es/ing/FichaLibro.php?Libro=331

[B20] Ruiz H Pavón JA (1798b) Systema vegetabilium florae peruvianae et chilensis. Typis Gabrielis de Sancha, Madrid. http://bibdigital.rjb.csic.es/ing/FichaLibro.php?Libro=107

[B21] Ruiz H Pavón JA (1799) Flora peruviana, et chilensis, Tomus II. Typis Gabrielis de Sancha, Madrid. http://bibdigital.rjb.csic.es/ing/FichaLibro.php?Libro=332

[B22] Ruiz H Pavón JA (1802) Flora peruviana, et chilensis, Tomus III. Typis Gabrielis de Sancha, Madrid. http://bibdigital.rjb.csic.es/ing/FichaLibro.php?Libro=333

[B23] Steele A (1964) Flowers for the King: The Expedition of Ruiz and Pavón and the Flora of Peru. Duke University Press, Durham.

[B24] Tafalla Navascués JJ (1989) Flora Huayaquilensis: sive descriptiones et icones plantarum huayaquilensium secumdum systema linnaeanum digestae; Tomus I. ICONA and Real Jardín Botánico, Madrid. http://bibdigital.rjb.csic.es/ing/FichaLibro.php?Libro=3967

[B25] Tafalla Navascues JJ (1991) Flora Huayaquilensis: sive descriptiones et icones plantarum huayaquilensium secundum systema linnaeanum digestae; Tomus II, icones. ICONA and Real Jardín Botánico, Madrid. http://bibdigital.rjb.csic.es/ing/FichaLibro.php?Libro=3968

[B26] Tepe EJ Bohs L (2011) A revision of SolanumsectionHerpystichum. Systematic Botany 36(4): 1068–1087. 10.1600/036364411X605074

[B27] Ulloa Ulloa C Acevedo Rodríguez P Beck S Belgrano MJ Bernal R Berry PE Brako L Celis M Davidse G Forzza RC Gradstein SR Hokche O León B León-Yánez S Magill RE Neill DA Nee M Raven PH Stimmel H Strong MT Villaseñor JL Zarucchi JL Zuloaga FO Jørgensen PM (2017) An integrated assessment of the vascular plant species of the Americas. Science 358(6370): 1614–1617. 10.1126/science.aao0398 29269477

